# Cell type‐specific gene expression changes are associated with cerebral amyloid angiopathy and blood‐brain barrier integrity in Alzheimer's disease

**DOI:** 10.1002/alz70855_106658

**Published:** 2025-12-24

**Authors:** Wei Tsai, Ozkan Is, Stephanie R Oatman, Tulsi Patel, Xue Wang, Joseph S. Reddy, Mariet Allen, Zachary Quicksall, Frederick Q Tutor‐New, Evan Udine, Shu Lin, Troy Carnwath, Kaancan Deniz, Thuy T. Nguyen, Michael G. Heckman, Chia‐Chen Liu, Yu Yamazaki, Yuka A Martens, Na Zhao, Minerva M. Carrasquillo, Melissa E. Murray, Guojun Bu, Takahisa Kanekiyo, Dennis W. Dickson, Nilüfer Ertekin‐Taner

**Affiliations:** ^1^ Mayo Clinic, Jacksonville, FL, USA; ^2^ Equal contribution, Jacksonville, FL, USA; ^3^ Icahn School of Medicine at Mount Sinai, New York, NY, USA; ^4^ University of Iowa, Iowa City, IA, USA; ^5^ Mayo Clinic in Florida, Jacksonville, FL, USA

## Abstract

**Background:**

More than 85% of Alzheimer's disease (AD) donor brains exhibit some degree of cerebral amyloid angiopathy (CAA), which is characterized predominantly by Aβ40 deposits in the brain vasculature and can cause blood brain barrier (BBB) leakage. CAA is associated with risk of infarcts, cerebral hemorrhages, and cognitive decline. Brain cell type‐specific mechanisms that influence CAA pathology, as well as related measures such as Aβ40 biochemical measures and tight junction proteins, an indicator of BBB integrity, are still elusive.

**Method:**

We performed single‐nucleus RNA sequencing (snRNAseq) of temporal cortex tissue from 79 AD donors, with varying levels of CAA co‐pathology. Measurements of soluble, insoluble and membrane‐associated fractions of brain Aβ40 and tight junction protein (claudin‐5, occludin) levels were available for these donors. We correlated these measures with cell proportions. We also conducted differential gene expression and analyses for intercellular communications and regulatory networks.

**Result:**

We identified 25 clusters and annotated them with major brain cell types. There were 3 oligodendrocyte, 7 excitatory and 7 inhibitory neuronal, 2 microglial, 2 astrocytic, and 1 each for endothelial, pericytic, fibroblast, and oligodendrocyte precursor cell clusters. We found that higher CAA and Aβ40 levels correlate with reduced neuronal and increased microglial, astrocytic and vascular cell proportions. Increased tight junction proteins correlate with increased inhibitory neuronal and reduced microglial, astrocytic and vascular cell proportions. Among all clusters, one microglial, both astrocytic and the pericytic clusters have greater number of genes significantly associated with CAA, Aβ40 and tight junction protein levels. Most significant genes have negative associations with CAA and membrane‐bound Aβ40 and positive with tight junction protein measures. Using these genes as input for intercellular communications analysis, we prioritized ligand‐target interactions from microglia or astrocytes to pericytes. Some of the prioritized targets are transcription factors in pericytes. Using regulon analysis, we identified genes downstream of these transcription factors that are also significantly associated with lower CAA and membrane‐bound Aβ40 and higher tight junction protein levels.

**Conclusion:**

We revealed glia‐to‐pericytes intercellular interactions and pericytic gene regulatory networks that are potentially perturbed by CAA and BBB breakdown. Future directions include analyzing external snRNAseq datasets and performing functional validations.

